# Burnout syndrome among preschool teachers in Serbia

**DOI:** 10.2478/aiht-2024-75-3825

**Published:** 2024-06-29

**Authors:** Pavle Piperac, Zorica Terzić-Supić, Aleksandra Maksimović, Jovana Todorović, Svetlana Karić, Ivan Soldatović, Smiljana Cvjetković, Vida Jeremić-Stojković, Simona Petričević

**Affiliations:** University of Belgrade, Faculty of Medicine, Department of Humanities, Belgrade, Serbia; University of Belgrade, Faculty of Medicine, Department of Social Medicine, Belgrade, Serbia; University of Kragujevac, Faculty of Science, Kragujevac, Serbia; Academy of Professional Studies, Department of Studies for Preschool and Nursery Teachers, Šabac, Serbia; University of Belgrade, Faculty of Medicine, Department of Medical Statistics and Informatics, Belgrade, Serbia; University Hospital Centre Bezanijska Kosa, Belgrade, Serbia

**Keywords:** Beck Depression Inventory, Copenhagen Burnout Inventory, demographics, kindergarten, lifestyle, mental health, Zung Self-Rating Anxiety Scale, Beckov upitnik o depresiji, Kopenhaški upitnik o izgaranju, demografija, dječji vrtić, način života, mentalno zdravlje, Zungova ljestvica za samoprocjenu simptoma anksioznosti

## Abstract

Pedagogical work, especially with preschool children, is one of the most stressful professions, and the incidence of stress-related illnesses among preschool teachers is higher than in the general population. The aim of this cross-sectional study, conducted between October 2018 and April 2019, was to examine the prevalence of the burnout syndrome in a representative sample of 482 preschool teachers in Serbia and the factors associated with it. For this purpose, the participants completed a questionnaire composed of six sections: the socio-demographic and socio-economic characteristics, health and lifestyle characteristics, workplace and employment characteristics; Copenhagen Burnout Inventory (CBI); Beck Depression Inventory (BDI), and the Zung Self-Rating Anxiety Scale (SAS). The frequency of the total burnout was 27.1 %. The frequency of burnout on the CBI was 25.4 % for personal burnout, 27.0 % for work-related burnout, and 23.4 % for client-related burnout. Multivariate logistic regression analysis with total burnout as an outcome variable showed that being single (OR: 0.18; 95 % CI: 0.05–0.58), having poor (OR: 6.05; 95 % CI: 1.05–34.91), or average (OR: 3.60; 95 % CI: 1.57–8.25) self-rated health, not having didactic/play tools (OR: 2.71; 95 % CI: 1.21–6.04), having a higher score on the BDI (OR: 1.19; 95 % CI: 1.09–1.29) or SAS (OR: 1.10; 95 % CI: 1.03–1.18) was significantly associated with the total burnout among our participants. Our study shows the worryingly high prevalence of the burnout syndrome among preschool teachers in Serbia and points to its association with mental health issues, depression, and anxiety.

## Sažetak

Pedagoški rad, posebice s djecom predškolske dobi, jedno je od najstresnijih zanimanja. Učestalost bolesti povezanih sa stresom veća je među odgajateljima nego u općoj populaciji. Cilj ovoga presječnog ispitivanja, provedenog od listopada 2018. do travnja 2019., bio je ispitati prevalenciju sindroma izgaranja na reprezentativnom uzorku od 482 odgajatelja u Srbiji i s tim povezane čimbenike. Sudionici su ispunjavali upitnik sastavljen od šest dijelova: sociodemografske i socioekonomske karakteristike, karakteristike zdravlja i stila života, karakteristike radnog mjesta i zaposlenja; Kopenhaški upitnik o izgaranju (*Copenhagen Burnout Inventory*, krat. CBI); Beckov upitnik o depresiji (*Beck Depression Inventory*, krat. BDI) i Zungova ljestvica za samoprocjenu anksioznosti (*Zung Self-Rating Anxiety Scale*, krat. SAS). Učestalost ukupnog izgaranja bila je 27,1 %. Učestalost izgaranja na CBI-ju bila je 25,4% za osobno izgaranje, 27,0 % za izgaranje na poslu i 23,4 % za izgaranje povezano s klijentima. Multivarijatna logistička regresijska analiza s ukupnim izgaranjem kao ishodnom varijablom pokazala je da su biti samac/samica (OR: 0,18; 95 % CI: 0,05–0,58), loše ili prosječno zdravlje (OR: 6,05; 95 % CI: 1,05–34,91 odnosno OR: 3,60; 95 % CI: 1,57–8,25), neposjedovanje didaktičkih sredstava/sredstava za igru (OR: 2,71; 95 % CI: 1,21–6,04) te viša ocjena depresije (OR: 1,19; 95 % CI: 1,09–1,29) ili SAS (OR: 1,10; 95 % CI: 1,03–1,18) bili značajno povezani s ukupnim izgaranjem među našim sudionicima. Naše ispitivanje pokazuje zabrinjavajuće visoku prevalenciju sindroma izgaranja među odgajateljima u Srbiji i upozorava na njegovu povezanost s problemima mentalnog zdravlja, depresijom i anksioznošću.

The burnout syndrome is caused by prolonged exposure to work-related stress and characterised by feelings of exhaustion, mental distancing from work, resentment and cynicism toward work and career, and poorer performance (1). Its prevalence varies greatly and reaches 82.4 % in some professions (2,3,4). Healthcare and social workers, prison officers, lawyers, and teachers are among the most vulnerable groups (5,6,7,8,9,10,11), and the prevalence of the burnout syndrome in preschool teachers is generally higher than 50 %, depending on the instrument used (9,10,11).

The factors that lead to the burnout syndrome may be individual and environmental (5). Individual factors include socio-demographic characteristics (age, gender, marital status, educational level, previous work experience), personality traits, and attitude towards work (too high job expectations) (6, 12). Environmental factors include job characteristics (working time, workload, shift work, poor working conditions, lack of work equipment, lack of autonomy in work and decision-making, lack of acknowledgment from superiors, and workplace conflicts) and client-related characteristics (client contacts, frequency of contacts, empathy, demanding clients) (5, 6). One of the individual factors associated with the burnout syndrome which has been most examined is gender, but the findings are inconsistent (13). Some studies show that men in predominantly female professions and women in predominantly male professions have a higher likelihood of the burnout syndrome (4, 8, 13, 14).

One of the most stressful professions is teaching, with preschool children in particular (7, 15). Over 60 % of preschool teachers claim that the leading sources of stress are excessive administrative work, crowded groups, small workplaces, and a lack of appropriate education materials (7). Preschool teachers constantly communicate with their supervisors, team colleagues (defectologists, pedagogues, psychologists, dentists, language teachers, other educators), and parents and are expected to meet their expectations (16). Also, they work in programmes for gifted children, programmes for foreign language learning, other artistic and sports activities, and rehabilitation programmes for children with special needs, all of which can be stressful (16, 17). Considering that pedagogical work involves different age groups (18), each age group has particular challenges as children learn to adopt healthy lifestyles and identify themselves as social beings (16, 19, 20).

The burnout syndrome among teachers has been studied and recognised in many countries as a public health issue (9, 10). In the South-East Europe, research on burnout syndrome among preschool teachers was conducted in Croatia, Greece, and Romania (7, 16, 21, 22) and showed high prevalence associated with age and years of work experience.

In Serbia, the burnout syndrome has been studied for more than two decades in different professions (4, 23,24,25,26,27), predominantly in healthcare workers (28,29,30,31). However, to the best of our knowledge, no such study has investigated burnout among preschool teachers in Serbia on the national level. The aim of this study was therefore to address this gap by establishing its prevalence and the factors associated with it.

## PARTICIPANTS AND METHODS

This cross-sectional study was conducted among preschool teachers in Serbia between October 2018 and April 2019 and was approved by the Ethics Committee of the University of Belgrade Medical Faculty (approval No. 1550/XI-49). It includes a sample of 482 participants, representative of the preschool teachers nationwide. To identify the 50 % burnout rate with the confidence interval of 95 % and precision of 5 % the sample required 401 participants as determined from the number of registered preschool teachers in Serbia (32). Our sample was divided into four clusters representing four statistical regions in Serbia: Belgrade, Northern Serbia, Western and Central Serbia, and Southern and Eastern Serbia. The size of each cluster was proportional to the number of preschool teachers in each region (32): 129 participants for Belgrade, 111 for Northern Serbia, 96 for Western and Central Serbia, and 65 for Southern and Eastern Serbia. The preschools were selected randomly from each cluster, and their number determined based on the average number of teachers per preschool per region. The study included all the preschool teachers who were at the workplace in the morning shift on the day of the study. All participants were given oral and written information about the study procedure and aims. We considered that all participants who filled in the questionnaire gave consent for participation.

The research instrument consisted of six sections combined in a single questionnaire: 1) socio-demographic and socio-economic characteristics, 2) health and lifestyle characteristics, 3) workplace and employment characteristics; 4) Serbian translation of the Copenhagen Burnout Inventory (26, 33, 34); 5) Serbian translation of the Beck Depression Inventory (35, 36), and 6) Serbian translation of the Zung Self-Rating Anxiety Scale (SAS) (37, 38).

The first three sections of the questionnaire were developed based on the questionnaires used in similar research (39,40,41,42,43). Self-perceived financial status was assessed with the question “How would you describe your financial status?” (very good/good/average/poor/very poor). We then merged the categories of “very good” and “good” into one – “good”, and the categories “poor” and “very poor” into one category – “poor”, whereas the category “average” remained.

Participant health characteristics were assessed with the following questions: “How would you describe your health status?” (very good / good/average / poor / very poor, merged as described above); “Did you take a sick leave (except for pregnancy, maternity leave, or sick leave due to a third party) in the past year?” (Yes / No); “If you took sick leave in the past year, how many days did it last?” (open-ended).

Lifestyle characteristics included smoking and alcohol consumption. Smoking status was assessed with the following questions: “Have you ever smoked tobacco?” [current smokers / ex-smokers (who used to smoke but had not smoked for more than 12 months) / non-smokers]; “Have you ever used electronic cigarettes?” (Never / Yes, but not anymore / Yes, I do now), whereas alcohol consumption and binge drinking were assessed with the following questions: “Did you consume any alcoholic beverages in the past year?” (Yes / No) and “Did you consume more than five drinks on one occasion (>1500 mL of beer, or 700 mL of wine, or 150 mL of spirits) in the past year?” (No / Yes, at least once in the past year but not in the past month / Yes, in the past month).

Workplace and employment characteristics were assessed with the following questions: “Do you have a tenured contract?” (Yes / No); “How many years of work experience do you have in total?” (open-ended); “How long have you been working at your current job?” (open-ended); “What is the age group of children you work with?” (three-four year-olds / five-six year-olds / school preparing program); “How many children are in the group you work with?” (open-ended); “Are there children with disabilities in the group you work with?” (Yes / No); “If there are children with disabilities in your group, how many are there?” (open-ended); “Does a child with a disability have a personal companion who is staying with them in the preschool?” (Yes / No); “Do you work in a central facility / branch)?”; “Do you have a managerial function?” (Yes / No); “Do you work in shifts?” (Yes / No); “Do you work overtime?” (Yes / No); “Do you have enough large and well-kept classrooms?” (Yes / No); “Do you have enough didactic/play tools?” (Yes / No); “Do you have a gym?” (Yes / No); “How much time do your administrative tasks take per day?” (<30 min / 30–60 min / >60 min); “How long is your commute from home to work?” (<30 min / 30–60 min / >60 min); “What type of transportation do you use on your way to work?” (public transport / car / bicycle/I walk).

The Copenhagen Burnout Inventory (CBI) (33) was used to assess burnout in three domains: personal (six questions), work-related (seven questions), and client-related burnout (six questions). The term “client” refers to those with whom employees come in contact at their workplace. In this study, these are children. The Serbian version of the CBI has been validated for use in preschool teachers (26), and the answers are given on a five-point Likert scale (0 – never; 1 – rarely; 2 – sometimes; 3 – often; 4 – always). Each point on the Likert scale represents the percentage of time (0 – 0 %; 1 – 25 %; 2 – 50 %; 3 – 75 %; 4 – 100 %), and the burnout score on each domain is the average percentage of the answers given in that domain. The total burnout is the average score of the three scales (26, 44). The Cronbach alphas for the individual scale in the Serbian version of CBI were 0.906 for personal burnout; 0.765 for work-related burnout; and 0.901 for client-related burnout. The test-retest reliability was 0.754 (intraclass correlation coefficient, ICC). All participants with the score above 50 are considered to have the burnout syndrome (44). All participants with the score above 50 on each scale are considered to have burnout in that domain (26).

We used the 21-item, self-rated scale Beck Depression Inventory (BDI) (36) to evaluate key symptoms of depression in preschool teachers, including mood, pessimism, sense of failure, self-dissatisfaction, guilt, punishment, self-accusation, suicidal ideas, irritability, social withdrawal, indecisiveness, body image change, difficulty working, insomnia, loss of appetite, weight loss, and loss of libido. Individual scale items are scored on a four-point scale with a total score ranging from 0 to 63. Higher scores indicate a greater likelihood of depression. The Cronbach alpha for BDI in our study was 0.849, ICC=0.927.

The Zung Self-Rating Anxiety Scale (SAS) (37) used in our study consists of 20 questions to measure anxiety. The answers are given on a four-point Likert scale (1 – never or rarely; 2 – sometimes; 3 – often; 4 – always). The total score ranges from 20 to 80, with the 20–44 score denoting normal range, 45–59 mild to moderate anxiety, 60–74 severe anxiety, and ≥75 extreme anxiety levels. The Cronbach alpha for SAS was 0.799, ICC=0.705.

### Statistical analysis

The statistical analyses run on the Statistical Package for Social Sciences (SPSS) version 22.0 (IBM Corp., Armonk, NY, USA). The comparisons between the groups with and without burnout were done using the chi-square and *t*-tests. All variables which showed significant differences were entered in the multivariate logistic regression model with total burnout as the outcome variable. The results were considered significant if P<0.05.

## RESULTS

The frequency of total burnout was 27.1 %. Personal burnout was 25.4 %, work-related burnout 27.0 %, and client-related burnout 23.4 %.

At the time of completing the survey, the dominant majority of participants were female (96.4 %), with an average age of 39.5±8.2 years. Most were married (67.5 %) and had children (72.6 %). Less than half of the participants (42.3 %) had university education, and two-thirds owned their apartment [323 (67.3 %)]. About a quarter reported poor [111 (23.2 %)] or good [117 (24.5 %)] financial status (Table 1).

**Table 1:** Socio-demographic and socio-economic characteristics of preschool teachers in Serbia (N=482)

**Characteristics**	**Total N (%)**	**With total burnout N (%)**	**Without total burnout N (%)**	**P-value**
**Gender**				
Male	17 (3.6)	2 (15.4)	11 (84.6)	
Female	458 (96.4)	119 (27.8)	309 (72.2)	0.323
**Age (mean±SD)**	39.5±8.2	43.9±7.8	38.9±9.5	**<0.001**
**Residence**				
Urban	328 (85.2)	87 (26.5)	241 (73.5)	
Rural	57 (14.8)	12 (21.1)	45 (78.9)	0.383
**Marital status**				
Married	318 (67.5)	95 (31.9)	203 (68.1)	
Single	153 (32.5)	24 (17.1)	116 (82.9)	**<0.001**
**Having children**				
Yes	349 (72.6)	105 (32.4)	219 (67.6)	
No	132 (27.4)	15 (12.3)	107 (87.7)	**<0.001**
**Years of education**				
≤15	274 (57.7)	71 (27.6)	186 (72.4)	
>15	201 (42.3)	50 (27.0)	135 (73.0)	0.889
**Owning an apartment**				
Yes	323 (67.3)	91 (30.1)	211 (69.9)	
No	157 (32.7)	30 (20.7)	115 (79.3)	**0.035**
**Self-rated financial status**				
Poor	111 (23.2)	39 (38.2)	63 (61.8)	
Average	250 (52.3)	63 (27.3)	168 (72.7)	
Good	117 (24.5)	19 (17.1)	92 (82.9)	**0.003**

More than half (58.6 %) reported good health, while 28.0 % were on sick leave in the past year, and the average length of sick leave was 16.7±25.6 days. Twenty-seven (6.3 %) reported heavy episodic drinking in the past month (Table 2).

**Table 2:** Health and lifestyle characteristics of preschool teachers in Serbia (N=482)

**Characteristics**	**Total N (%)**	**With total burnout N (%)**	**Without total burnout N (%)**	**P-value**
**Self-rated health**				
Poor	20 (4.9)	10 (71.4)	4 (28.6)	
Average	150 (36.5)	64 (44.1)	81 (55.9)	
Good	241 (58.6)	24 (10.7)	201 (89.3)	**<0.001**
**Sick leave in the past year**				
Yes	132 (28.0)	45 (35.7)	81 (64.3)	
No	339 (72.0)	73 (23.5)	238 (76.5)	**0.009**
**Number of days of sick leave (mean±SD)**	16.7±25.6	21.1±25.2	18.3±30.3	**<0.001**
**Smoking**				
Yes	158 (33.1)	38 (26.0)	108 (74.0)	
No	319 (66.9)	83 (28.0)	213 (72.0)	0.655
**Ever smoked electronic cigarettes**				
Yes	22 (5.2)	8 (40.0)	12 (60.0)	
No	394 (92.5)	88 (23.9)	280 (76.1)	
Used to, but stopped	10 (2.3)	3 (33.3)	6 (66.7)	0.227
**Alcohol consumer**				
Yes	299 (62.7)	76 (27.2)	203 (72.8)	
No	178 (37.3)	44 (26.7)	121 (73.3)	0.895
**Binge drinking**				
No	378 (88.7)	95 (27.0)	257 (73.0)	
Yes, in a past year, but not in the past month	21 (4.9)	11 (55.0)	9 (45.0)	
Yes, in the past month	27 (6.3)	4 (16.0)	21 (84.0)	**0.010**
No	224 (88.9)	64 (30.3)	147 (69.7)	0.055

The average total work experience was 14.5±7.8 and the average work experience on a current job was 9.6±6.2 years. Around three-quarters of the participants (75.2 %) had tenured contracts. The average number of children per group was 26.1±5.3. One-third (30.3 %) reported not having appropriate didactic/play tools to work with (Table 3).

**Table 3:** Workplace and employment characteristics of preschool teachers in Serbia (N=482)

**Characteristics**	**Total N (%)**	**With total burnout N (%)**	**Without total burnout N (%)**	**P-value**
**Tenured contract**				
Yes	358 (75.2)	105 (31.3)	230 (68.7)	
No	118 (24.8)	14 (13.1)	93 (86.9)	**<0.001**
**Work experience (mean±SD)**	14.5±7.8	18.9±8.7	13.4±9.1	**<0.001**
**Work experience on a current job (mean±SD)**	9.5±6.2	14.1±9.7	9.9±8.7	**<0.001**
**Age of the children in group**				
three – four years	207 (43.3)	53 (27.5)	140 (72.5)	
five – six years	132 (27.6)	29 (23.4)	95 (76.6)	
school preparing program	89 (18.6)	30 (37.0)	51 (63.0)	
Other	50 (10.5)	9 (20.0)	36 (80.0)	0.110
**Number of children per group (mean±SD)**	26.1±5.3	26.9±5.7	25.3±6.5	**0.018**
**Children with disability in the group**				
Yes	201 (42.4)	56 (29.6)	133 (70.4)	
No	273 (57.6)	63 (25.1)	188 (74.9)	0.290
**Number of children with disability per group (mean±SD)**	1.8±1.4	1.8±1.4	1.6±1.1	0.225
**Personal accompany for children with disability**				
Yes	28 (11.1)	3 (12.0)	22 (88.0)	
No	224 (88.9)	64 (30.3)	147 (69.7)	0.055
**Workplace location**				
Centrally located	338 (83.7)	85 (26.8)	232 (73.2)	
Peripherally located	66 (16.3)	9 (15.8)	48 (84.2)	0.077
**Managerial function**				
Yes	56 (11.7)	12 (22.2)	42 (77.8)	
No	424 (88.3)	108 (27.6)	283 (72.4)	0.402
**Shift work**				
Yes	406 (84.6)	108 (28.5)	271 (71.5)	
No	74 (15.4)	13 (19.4)	54 (80.6)	0.123
**Overtime**				
Yes	116 (24.6)	33 (30.0)	77 (70.0)	
No	356 (75.4)	87 (26.4)	243 (73.6)	0.458
**Enough large and well-kept rooms**				
Yes	338 (71.0)	78 (24.5)	240 (75.5)	
No	138 (29.0)	41 (33.1)	83 (66.9)	0.156
**Didactic/play tools**				
Yes	325 (69.7)	65 (21.6)	236 (78.4)	
No	141 (30.3)	52 (39.4)	80 (60.6)	**<0.001**
**Gym**				
Yes	237 (50.7)	60 (26.8)	164 (73.2)	
No	230 (49.3)	56 (26.7)	154 (73.3)	0.978
**Time for administrative work per day**				
<30 min	146 (31.5)	33 (25.6)	96 (74.4)	
30–60 min	272 (58.6)	68 (26.3)	191 (73.7)	
>60 min	46 (9.9)	16 (36.4)	28 (63.6)	0.340
**Commute time**				
<30 min	296 (62.3)	72 (26.3)	202 (73.7)	
30–60 min	126 (26.5)	31 (35.6)	90 (74.4)	
>60 min	53 (11.2)	17 (34.0)	33 (66.0)	0.488
**Type of transportation**				
Public transportation	141 (34.4)	37 (27.2)	99 (72.8)	
Car	111 (27.1)	18 (17.5)	85 (82.5)	
Bicycle	17 (4.1)	4 (25.0)	12 (75.0)	
Walking	141 (34.4)	37 (29.1)	90 (70.9)	0.203

The average Beck Depression Inventory score was 4.4±5.1; 8.8±6.1 in participants with the total burnout score above the 50-point threshold, and 2.9±3.8 in those with the total burnout below the 50-point threshold (P<0.001).

The average score on the Zung Self-Rating Anxiety Scale was 32.5±7.1; 38.2±7.3 for participants above the total burnout threshold and 30.4±5.8 for participants below it (P<0.001) (Table 4).

**Table 4:** Mental health characteristics of preschool teachers in Serbia (N=482)

**Characteristics**	**Total (mean±SD)**	**With total burnout (mean±SD)**	**Without total burnout (mean±SD)**	**P-value**
**Beck Depression Inventory score**	4.4±5.1	8.8±6.1	2.9±3.8	**p<0.001**
**Zung Self-Rating Anxiety Scale score**	32.5±7.1	38.2±7.3	30.4±5.8	**p<0.001**

Multivariate logistic regression analysis with total burnout as an outcome variable shows that it was significantly associated with being single (OR: 0.18; 95 % CI: 0.05–0.58), having poor (OR: 6.05; 95 % CI: 1.05–34.91) or average (OR: 3.60; 95 % CI: 1.57–8.25) self-rated health, not having didactic/play tools (OR: 2.71; 95 % CI: 1.21–6.04), having a higher score on the Beck Depression Inventory (OR: 1.19; 95 % CI: 1.09–1.29) or Zung Self-Rating Anxiety Scale (OR: 1.10; 95 % CI: 1.03–1.18) (Table 5).

**Table 5:** Multivariate logistic regression analysis with total burnout as an outcome variable

**Characteristics**	**OR (95 % CI)**
**Age (mean±SD)**	0.98 (0.89–1.08)
**Marital status**	
Married	1.0 (reference category)
Single	**0.18 (0.05–0.58)**
**Having children**	
Yes	1.0 (reference category)
No	1.39 (0.37–5.20)
**Owning an apartment**	
Yes	1.0 (reference category)
No	0.96 (0.37–2.53)
**Tenured contract**	
Yes	1.0 (reference category)
No	0.60 (0.19–1.86)
**Work experience (mean±SD)**	1.03 (0.93–1.14)
**Work experience on a current job (mean±SD)**	1.03 (0.98–1.10)
**Self-rated financial status**	
Poor	3.33 (0.99–11.14)
Average	2.35 (0.83–6.64)
Good	1.0 (reference category)
**Self-rated health**	
Poor	**6.05 (1.05–34.91)**
Average	**3.60 (1.57–8.25)**
Good	1.0 (reference category)
**Binge drinking**	
No	1.0 (reference category)
Yes, in a past year, but not in the past month	5.63 (0.74–42.57)
Yes, in the past month	3.05 (0.68–13.57)
**Sick leave in the past year**	
Yes	0.92 (0.40–2.11)
No	1.0 (reference category)
**Number of children per group (mean±SD)**	1.04 (0.98–1.10)
**Didactic/play tools**	
Yes	1.0 (reference category)
No	**2.71 (1.21–6.04)**
**Beck Depression Inventory score**	**1.19 (1.09–1.29)**
**Zung Self-Rating Anxiety Scale score**	**1.10 (1.03–1.18)**

## DISCUSSION

Our study shows that total burnout, established in 27.1 % of our participants is associated with being single, having poor or average self-rated health, not having didactic/play tools available at work, and scoring high on the Beck Depression Inventory and Zung Self-Rating Anxiety Scale. It is lower than the prevalence reported among Chinese preschool teachers (53.2 %) (11) and more similar to the one reported by preschool teachers in Iraq (45).

Our study also confirms earlier reports that the burnout syndrome is associated with depression (10, 11). In our sample, every point on the Beck Depression Inventory corresponds to a 19 % increase in the likelihood of total burnout. Some authors (11, 46) have argued that the association between depression and burnout may be owed to personality traits such as neuroticism. These individuals may be more prone to a higher stress reaction and more negative emotions. However, this association may be biased, as many individuals with the burnout syndrome meet the diagnostic criteria for depression, and burnout may manifest as depression (47,48,49).

The burnout syndrome in our study is also associated the score on the Zung Self-Rating Anxiety Scale, and each point on this scale corresponds to a 10 % increase in the likelihood of total burnout. Respondents with higher levels of anxiety may feel more stressed (10, 50), and higher levels of perceived stress have been shown to affect personal autonomy, feeling of competence, and relatedness, all of which increase the risk of the burnout syndrome (10, 11).

Additionally, burnout established in our sample is associated with six times higher likelihood of reporting poor self-rated health and more than 3.5 times higher likelihood of reporting average health than to reporting good health. These findings may reflect higher likelihood of depression and anxiety, as both conditions are associated with poorer self-rated health (51,52,53,54). The burnout syndrome is also associated with higher levels of musculoskeletal pain and cardiovascular diseases, which also contributes to poorer self-rated health (55, 56).

Even though work with special needs children (17, 57, 58) is often identified as cause of stress among preschool teachers, we found no such association in our study. Similar findings have been reported in a Turkish study (57), which showed that having integration children does not increase burnout among preschool teachers.

Our study shows a five times lower likelihood of burnout among single preschool teachers, whereas earlier studies report inconsistent findings in this respect (59,60,61,62). As for having children, we found no association with burnout, either positive or negative, whereas some report that having children has a protective effect against it, as preschool teachers with their own children easily establish a relationship with children, love their work better, and work more efficiently (63).

## CONCLUSION

Our study shows a worryingly high prevalence of the burnout syndrome among preschool teachers in Serbia and its association with poor (self-rated) health and mental health issues, depression and anxiety in particular.

It points to a need for regular health and burnout assessments and the implementation of specific preventive measures, starting with setting up a healthy workplace environment.

The study’s main limitation lies in its cross-sectional design, which cannot establish the causal relationships between the variables. Another limitation is that health, including mental health scores are self-reported, so some symptoms may be under- or overestimated. The main strength is in its large and nationally representative sample from urban and rural environments. This is, to the best of our knowledge, the first study on the burnout syndrome conducted on this population in Serbia, and its results can be valuable for policymakers and professionals developing preventive programmes.
